# Construction of ceRNA prognostic model based on the CCR7/CCL19 chemokine axis as a biomarker in breast cancer

**DOI:** 10.1186/s12920-023-01683-9

**Published:** 2023-10-21

**Authors:** Rufei Ma, Xiuliang Guan, Nan Teng, Yue Du, Shu Ou, Xiaofeng Li

**Affiliations:** Department of Epidemiology, Dalian Medical University, Dalian, China

**Keywords:** CCR7, CCL19, ceRNA network, Breast cancer, Immunity, Cell migration ability

## Abstract

**Background:**

The study of CCR7/CCL19 chemokine axis and breast cancer (BC) prognosis and metastasis is a current hot topic. We constructed a ceRNA network and risk-prognosis model based on CCR7/CCL19.

**Methods:**

Based on the lncRNA, miRNA and mRNA expression data downloaded from the TCGA database, we used the starbase website to find the lncRNA and miRNA of CCR7/CCL19 and established the ceRNA network. The 1008 BC samples containing survival data were divided into Train group (504 cases) and Test group (504 cases) using R “caret” package. Then we constructed a prognostic risk model using RNA screened by univariate Cox analysis in the Train group and validated it in the Test and All groups. In addition, we explored the correlation between riskScores and clinical trials and immune-related factors (22 immune-infiltrating cells, tumor microenvironment, 13 immune-related pathways and 24 HLA genes). After transfection with knockdown CCR7, we observed the activity and migration ability of MDA-MB-231 and MCF-7 cells using CCK8, scratch assays and angiogenesis assays. Finally, qPCR was used to detect the expression levels of five RNAs in the prognostic risk model in MDA-MB-231 and MCF-7 cell.

**Results:**

Patients with high expression of CCR7 and CCL19 had significantly higher overall survival times than those with low expression. The ceRNA network is constructed by 3 pairs of mRNA-miRNA pairs and 8 pairs of miRNA-lncRNA. After multivariate Cox analysis, we obtained a risk prognostic model: riskScore= -1.544 *`TRG-AS1`+ 0.936 * AC010327.5 + 0.553 *CCR7 -0.208 *CCL19 -0.315 *`hsa-let-7b-5p. Age, stage and riskScore can all be used as independent risk factors for BC prognosis. By drug sensitivity analysis, we found 5 drugs targeting CCR7 (convolamine, amikacin, AH-23,848, ondansetron, flucloxacillin). After transfection with knockdown CCR7, we found a significant reduction in cell activity and migration capacity in MDA-MB-231 cells.

**Conclusion:**

We constructed the first prognostic model based on the CCR7/CCL19 chemokine axis in BC and explored its role in immune infiltration, tumor microenvironment, and HLA genes.

**Supplementary Information:**

The online version contains supplementary material available at 10.1186/s12920-023-01683-9.

## Introduction

Breast cancer is a serious threat to women’s health and life in today’s society. According to statistics, about 1.3 million new cases of breast cancer (BC) occur each year worldwide, and 450,000 patients die from BC. Despite innovations and improvements in surgery, radiation and drug treatment techniques, BC incidence and mortality have not decreased significantly [1]. Currently, there are no specific tumor-related markers for BC. In the era of precision therapy, it is urgent to find molecular biomarkers that are closely related to the development of BC and provide new tools for BC diagnosis and treatment.

Chemokines are a class of small molecule cytokines with a molecular weight of 8–12 KDa. They are the largest subfamily of cytokine family and can bind to chemokine receptors for the chemotactic movement of cells. More than 50 kinds of chemokines and 20 kinds of chemokine receptors have been discovered so far. Chemokines can be further classified into CC, CXC, CX3C and XC subgroups based on the location of the N-terminal cysteine disability in their protein sequence [2]. CCR7 is a G protein-coupled receptor expressed on the membranes of naturally activated T cells, B cells and dendritic cells, and contains 378 amino acids (45 KDa) [3, 4]. CCR7 is expressed in both primary BC cells and metastatic BC cells and plays an important role in promoting the spread and migration of BC cells [5]. CCL19, also known as MIP-3β and exodus-3, is a chemokine expressed in secondary lymphoid organs and the thymus and contains 98 amino acids (8.8 KDa) [6, 7]. The CC chemokine receptor member CCR7 interacts with its ligands CCL19 and CCL21 to play an important role in the growth and development of lymphoid organs and the targeting of lymphocytes and dendritic cells to lymph nodes for homing.

Competitive endogenous RNA (ceRNA) hypothesis is a new regulation mode of gene expression. Its theory mainly refers to that long non-coding RNA (lncRNA) and Circular RNA (circRNA) can competitively bind MicroRNA (miRNA), interfere with the binding of miRNA and Messenger RNA (mRNA), thus regulating gene expression and affecting cell function [8]. Although ceRNA network has been verified in a variety of tumor cells, the ceRNA network of CCR7/CCL19 chemokine axis in BC has not been studied. In this study, we constructed a CCR7/CCL19 ceRNA network and predictive risk model based on TCGA database and some online websites. Meanwhile, we analyzed the relationship between riskScore of predictive risk model and some factors of immune.

## Materials and methods

### Data download and collation

We downloaded transcriptome RNA-sequencing data of count file of lncRNA, miRNA, and mRNA and clinical information of BRCA patients the Cancer Genome Atlas (TCGA) database (https://tcga-data.nci.nih.gov/tcga/). For the transcriptome data, we downloaded human.gtf from Ensembl (http://asia.ensembl.org/index.html), which was used to Transform Ensembl_ID into gene names through Perl scripts. For miRNA data, mature.fa obtained from miRbase (http://microrna.sanger.ac.uk/) transform miRNA sequences into human mature miRNA names through Perl scripts. Finally, the transcriptome data included 1208 samples (112 normal samples and 1096 tumor samples), miRNA data included 1193 samples (103 normal samples and 1090 tumor samples), and clinical data included 1085 samples.

### Difference and survival analysis of CCR7 and CCL19

According to TCGA sample barcode rule, we extracted paired samples and CCR7 and CCL19 corresponding expression matrix. *We used 110 matched pairs of breast cancer and paracancerous tissues from the TCGA-BRCA data to perform pairwise difference analysis for CCR7 and CCL19*, and plotted boxplots using R “ggplot2” package. Meanwhile, CCR7 and CCL19 were analyzed using the Gene Expression Profiling Interactive Analysis (GEPIA) online tool (http://gepia.cancer-pku.cn/), which integrated gene expression profile data from TCGA and Genotype-Tissue Expression (GTEx) projects to add more expression data from normal samples [9]. The samples were divided into high-low expression groups according to the median value of gene expression in TCGA database, and the survival data were combined for Kaplan-Meier analysis with R “survival” package. In addition, Kaplan-Meier Plotter and GEPI online tools were used for verification. Finally, the “ggsurvplot” function was used to plot the survival curve of CCR7 and CCL19 combined grouping. In addition, we performed a meta-analysis combined with Cox analysis from the PrognoScan online tool (http://dna00.bio.kyutech.ac.jp/PrognoScan/) to investigate the relationship between CCR7 and CCL19 expression levels and prognosis.

### Establishment of ceRNA network

Based on the hypothesis that lncRNA competes with miRNA to regulate mRNA expression in the cytoplasm, we constructed the ceRNA network with the following steps: Firstly, starbase (https://starbase.sysu.edu.cn/) was used to find the CCR7 and CCL19 binding miRNAs respectively, and miRNA-mRNA co-expression analysis screened final targeted miRNA. Secondly, the same method as above was used to find the final miRNA-targeted lncRNA. Thirdly, cytoscape software constructs the ceRNA network and outputs the visualization results. *The criteria for screening were that IncRNAs showed the same expression trend as mRNAs and were opposite to miRNAs.* Since the expression of miRNA-mRNA and miRNA-lncRNA in the ceRNA network needed to meet the negative correlation relationship, spearman test was used to verify the association strength between RNAs with screening condition of Pearson correlation coefficient *r*<-0.15 and *p* value < 0.05. Starbase is an online database of RNA-RNA and RNA-protein interaction networks constructed based on 37 independent sequencing experiments [10], which contains the prediction results of 7 software. When two or more software predictions were met, the miRNA or lncRNA was included in the subsequent analysis.

### Construction of prognostic model

One thousand eight BRCA samples integrated with survival data were divided into Train group (504) and Test group (504) using R “caret” package. Multivariate Cox analysis was conducted for all mRNA, miRNA and lncRNA in the ceRNA network obtained from the above analysis screening out survival related RNAs *with a screening criterion of P < 0.01*. Risk assessment model was constructed according to the regression coefficient of each RNAs. BRCA samples from Train group, Test group and All group were divided into high and low-risk group according to the median riskScore of Train group. Survival difference between the two groups was compared using R “survival” and “survminer” package, and the survival curve was displayed. Receiver operating characteristic curve (ROC) curves and calibration curves for 1-, 3-, and 5-years were plotted using R “timeROC” and “rms” packages to evaluate the predictive performance of riskScores. PCA curve was drawn using R “ggplot2” package to compare the survival status of the high- or low-risk groups. Area Under Curve (AUC) measures the predictive power of risk models for clinical outcomes.

### Correlation with clinical trails

Univariate and multivariate Cox analyses were performed in riskScore and age and TNM stage extracted from TCGA clinical database. And nomogram was draw based on the result of multivariate Cox analysis with “rms” and “regplot” package. *As a two-dimensional functional image with coordinates, the Nomogram can visualize multiple predictors, quantify the prognostic risk of a disease, and develop an individualized and reliable reference for patients.* ROC curves and calibration curves of 1-,3- and 5- years were performed to evaluate the degree of consistency between the predicted results of nomograph model and the actual results.

### Enrichment analysis

To further determine the biological functions and pathway of risk model, we firstly set |logFC|>1 and FDR < 0.05 as threshold and performed risk difference analysis to obtain risk difference genes. Then, R “org.hs.eg. db” package was used for GO and KEGG enrichment analysis of risk differential genes, and the analysis results were summarized according to screening conditions (*P* < 0.05) presented as bubble charts. In addition, STRING (https://www.string-db.org/) and Cytoscape (version3.8.2) software were used to map the protein-protein interaction (PPI) network of risk differential genes. When using a STRING database, we set the correlation as 0.7. The top 10 genes were screened by cBioPortal plug-in.

### Correlation with immune-related factors

To explore the relationship between ceRNA risk model and some immune-related factors, we performed Wilcoxon rank-sum test and spearman correlation test with 22 immune-infiltrating cells, tumor microenvironment, 13 immune-related pathways and 24 HLA genes. The CIBERSORT algorithm were utilized to assess the 22 kinds of immune cell types and in BRCA with the threshold of *p* < 0.05. *The algorithm is an inverse convolutional algorithm to calculate the expression of immune cells infiltrated in 22 tumors.* The ESTIMATE algorithm was performed to quantify the tumor microenvironment, including Immunescore and Stromalscore. *The Estimate algorithm is a commonly used algorithm for immune infiltration analysis, which allows scoring of tumor stromal cell and immune cell infiltration results from transcriptome sequencing data or gene expression obtained from gene microarrays* [11]. 16 Immunoinfiltrating cells were scored by single-sample gene set enrichment analysis (ssGSEA) with R “gsva” package, and 13 immune-related pathways were analyzed [12]. Human leukocyte antigen (HLA) system is a closely linked gene group, which is the most complex polymorphic system in Human body.

### Drug sensitivity analysis in cMap

The up- or down-risk difference genes input connectivitymap (cMap) database (http://www.broad.mit.edu/cmap/) as Query Signature file format. And the risk differential genes were compared with the reference gene expression profile in cMap to find relevant small molecule compounds or drugs. The results were sorted by score to screen out small molecule compounds or drugs with negative correlation. Then molecular structure of the drug was found through PubChem online tool (https://pubchem.ncbi.nlm.nih.gov/).

### Cell transfection

The siCCR7 sequence (5’-CCAUCUACAAGAUGAGCUUTT-3’) was entrusted to Girma Genetics Ltd. The plasmid and lipofectamineTM3000 (Thermo Fisher Scientific, L3000015) were diluted separately with 200 µl of serum-free medium, then the two were mixed thoroughly and left to stand for 10 min to allow the plasmid to be fully encapsulated. After 6 h the supernatant was discarded and replaced with fresh DMEM medium for subsequent analysis.

### CCK-8 assay

After transfection, cells were digested with trypsin and inoculated in 96-well plates. 10 µL of CCK8 solution (Bioss, BA00208) was added to each well on day 2 to avoid air bubbles. The plates were incubated in an incubator for 2 h. We measured the absorbance (OD) at 450 nm using an enzyme marker and calculated the cell viability (cell viability= (OD_Experimental group_ -OD _Blank group_)/ (OD _Control group_ -OD _Blank group_) ×100%).

### Wound healing assay

We started by first making even horizontal lines on the back of the 6-well plate with a marker pen. Inoculate the cells in the 6-well plate at a density of 5 × 105. The next day a 200 µl gun was used to score the horizontal line perpendicular to the back with a straightedge, the gun should be vertical and not tilted. Wash the cells 3 times with PBS to remove the scratched down cells and add serum-free medium. Place in incubator and incubate. Remove the 96-well plate at 0 and 24 h to take pictures under the microscope respectively. And the results were quantified using imageJ software.

### Angiogenesis assay

Cells were inoculated onto Matrigel-lined 96-well plates and incubated in a cell culture incubator and observed under the microscope for lumen formation at 2, 6 and 12 h respectively. Microscopic photographs were taken, and the results were quantified using imageJ software.

### Quantitative real-time PCR

Total RNA was extracted from MCF-7 and MDA-MB-23 breast cancer cells (purchased from Wuhan Procell Life Science&Technology Co., Ltd.) using AG RNAex Pro Reagent (AG21101). Reverse transcription was performed using Evo M-MLV RT Kit with gDNA Clean for qPCR (AG11705). RT-PCR was performed using SYBR Green Premix Pro Taq HS qPCR Kit (AG11701) in Rotor-Gene Q instrument (Qiagen). Each sample was tested in triplicates, and each sample underwent a melting curve analysis to check for the specificity of amplification. The expression level was determined as a ratio between the hub genes and the internal control GAPDH or U6 in the same mRNA sample and calculated by the comparative Ct method. The relative expression of target genes was calculated by ΔΔCt method. The primer sequences are as Table 1.

**Table 1:** Primer sequences of components in ceRNA network

Gene	Forward (5’→3’)	Reverse (5’→3’)
GAPDH	GCACCGTCAAGGCTGAGAAC	TGGTGAAGACGCCAGTGGA
U6	GGAACGATACAGAGAAGATTAGC	TGGAACGCTTCACGAATTTGCG
CCR7	CCAGAGGAGCAGCAFTGA	CGGATGATCACAAGGTAACAGAAG
CCL19	GGTGCCTGCTGTAGTGTTC	AGTCTCTGGATGATGCGTTCTA
TRG-AS1	CTTAGAGCCTCCGTATCTGT	TTATGATGGCTACGATGTCAC
AC010327.5	CTCGCTCAGCTAACAGGA	GCTCCACCACAAACACT
hsa-let-7b-5p	CGCGTGAGGTAGTAGGTTGT	AGTGCAGGGTCCGAGGTATT

## Results

### Expression and prognostic value of CCR7 and CCL19

**Fig. 1:**
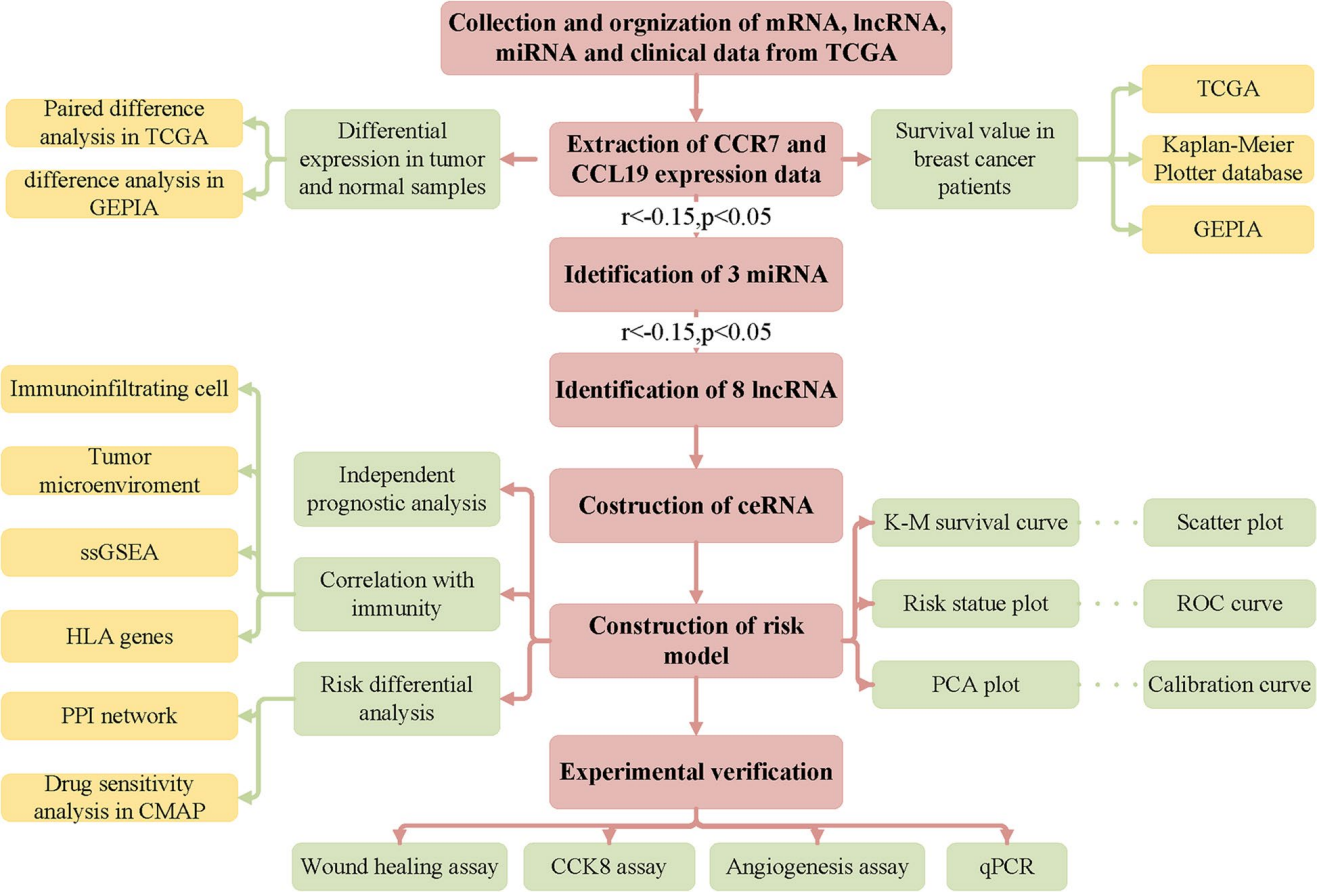
Flowchart of the analysis process

**Fig. 2:**
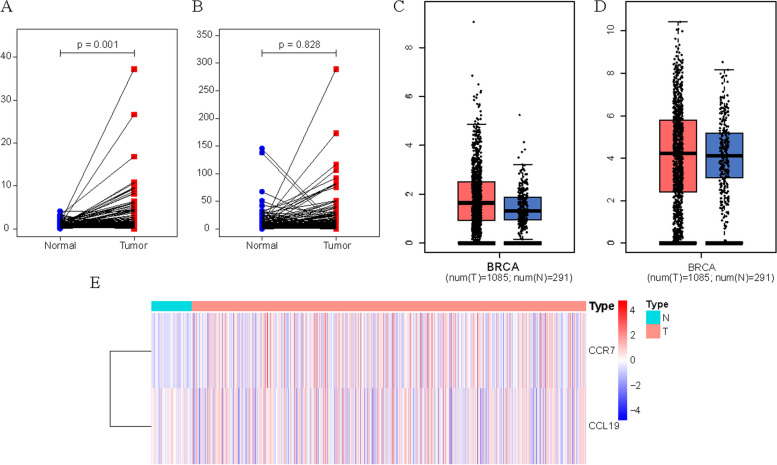
Differential expression of CCR7 and CCL19 in BC. **A** Pairing difference analysis of CCR7 in TCGA database. **B** Pairing difference analysis of CCL19 in TCGA database. **C** Difference analysis of CCR7 in GEPIA online tool. **D** Difference analysis of CCL19 in GEPIA online tool. **E** Difference heatmap of CCR7 and CCL19 in TCGA database

**Fig. 3:**
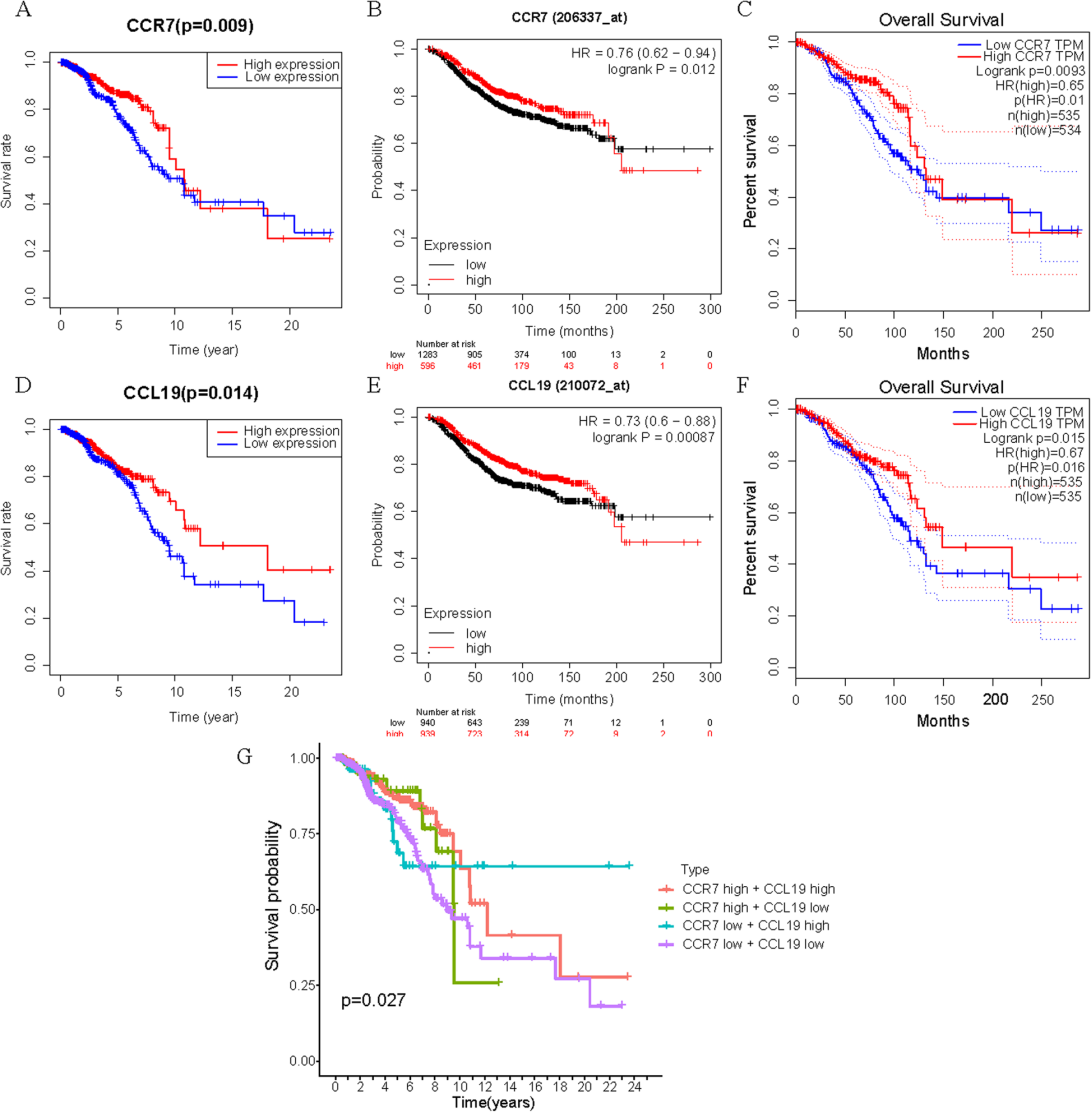
Kaplan-Meier survival curve of CCR7 and CCL19. **A**,** D** In TCGA database, the correlation between OS and CCR7 (A) or CCL19 (**D**) expression level. **B**,** E** In Kaplan-Meier Plotter online tool, the correlation between OS and CCR7 (**B**) or CCL19 (**E**) expression level. **C**,** F** In GEPIA online tool, the correlation between OS and CCR7 (C) or CCL19 (**F**) expression level. **G** Combined survival analysis of CCR7 and CCL19

Our analysis process is shown in Fig. 1. Initially, we analyzed the expression of CCR7 and CCL19 and their relationship with overall survival (OS) time in BC. On the one hand, we found that CCR7 was highly expressed in tumor samples in the TCGA database (*p* < 0.05), while the difference in CCL19 was not statistically significant (*p* = 0.328) (Fig. 2A, B). The same results were obtained in GEPIA online tools (Fig. 2C, D). Figure 2E *shows the lighter color of CCR7 in the TCGA-BRCA paracarcinoma samples, indicating a lower expression of CCR7.* On the other hand, in all of TCGA database (Fig. 3A, D), Kaplan-Meier Plotter (Fig. 3B, E) and GEPIA (Fig. 3C, F) online tools, K-M test results showed that patients with high expression of CCR7 or CCL19 had significantly higher overall survival (OS) than those with low expression of that (*p* < 0.05). Figure 3G shows that when the expression levels of CCR7 and CCL19 are both high, the survival rate of BC patients is the highest.

### Establishment of ceRNA network

**Fig. 4:**
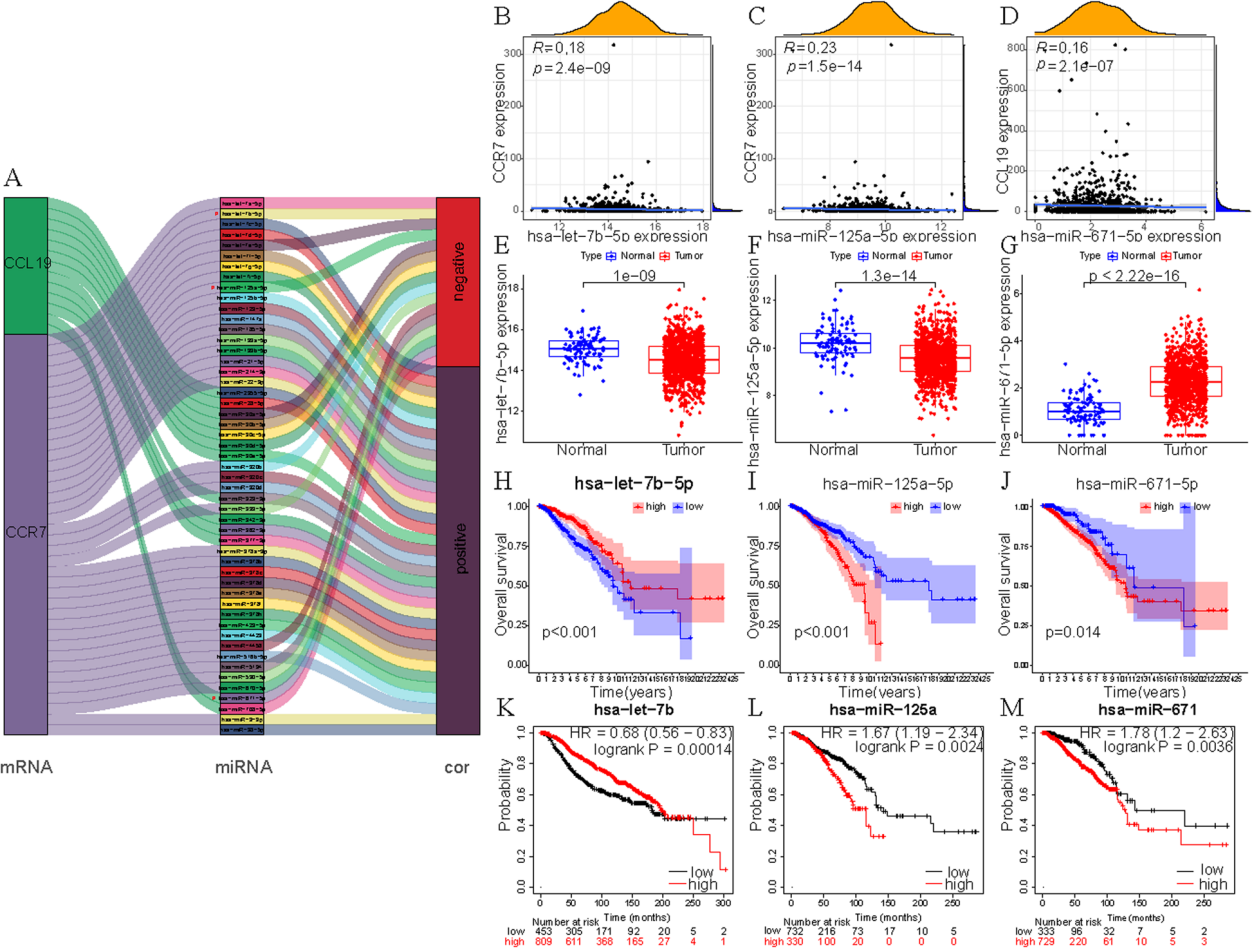
Construction of mRNA-miRNA. **A** Sankey diagram showed the association between CCR7, CCL19, miRNAs and risk type. **B**,** C** Correlation between CCR7 and has-let-7b-5p (**B**) and has-Mir-125a-5p in TCGA database (**C**). **D** Correlation between CCL19 and has-Mir-671-5p in TCGA database. **E-G** Differentially expressed has-let-7b-5p (**E**), has-Mir-125 A-5p (F) or has-Mir-671-5p (**G**) in TCGA database. **H-J** Correlation between OS and HAS-let-7b-5p (**H**), has-Mir-125 A-5p (**I**) or has-Mir-671-5p (**J**) in TCGA database. **K-M** Correlation between OS and Has-let-7b-5p (K), has-Mir-125 A-5p (**L**) or has-Mir-671-5p (**M**) in Kaplan-Meier Plotter online tool (COR&LT; 0.15). “#” represents the miRNAs whose correlation coefficient was less than − 0.15 in the mRNA-miRNA Spearman test and was included in subsequent analysis

We randomly divided the samples of BC patients into Train and Test groups in this study, we established a lncRNA-miRNA-mRNA ceRNA network. Firstly, according to starbase database, we downloaded 46 CCR7-miRNA pairs and 13 CCL19-miRNA pairs (Fig. 4A). Then three miRNAs were identified by mRNA-miRNA co-expression analysis (cor<-0.15, *p* < 0.05), including has-miR-125a-5p, has-let-7b-5p and has-miR-671-5p. They were all negatively correlated with the expression of CCR7 (Fig. 4B-D). has-let-7b-5p and has-miR-671-5p are highly expressed in tumor patients, while has-miR-125a-5p is opposite (Fig. 4E-G). In TCGA and Kaplan-Meier Plotter databases, we found that high expression of has-let-7b-5p was associated with higher overall survival, while low expression of has-miR-125a-5p and has-miR-671-5p were associated with higher OS (*p* < 0.05) (Fig. 4H-M).

**Fig. 5:**
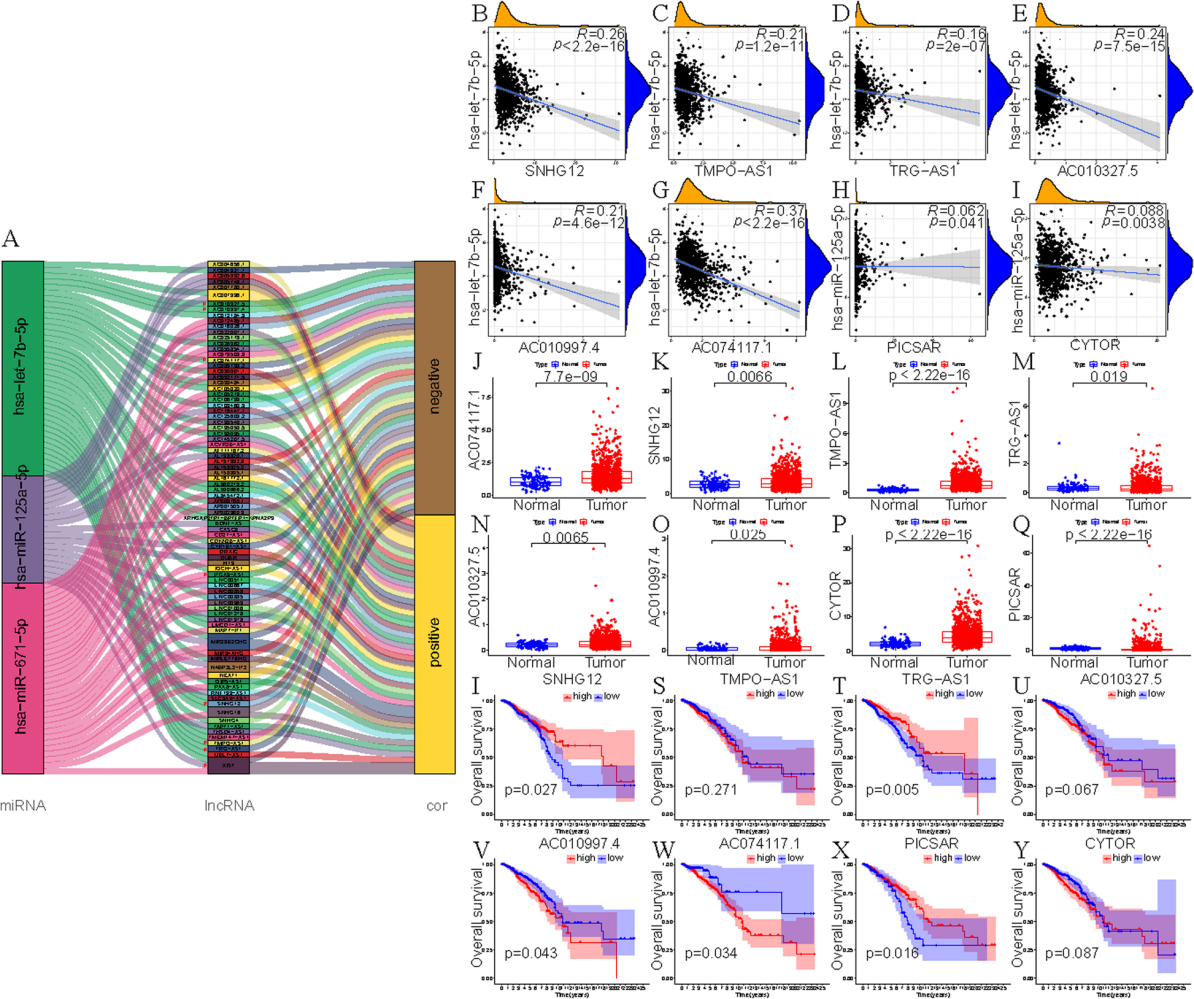
Construction of miRNA-lncRNA. **A** Sankey diagram showed the association between has-let-7b-5p, has-miR-125a-5p, has-miR-671-5p, lncRNAs and risk type (|cor|>0.1). **B**-**G** Correlation between has-let-7b-5p and targeted lncRNAs in TCGA database. **H**,** I** Correlation between has-miR-125a-5p and targeted lncRNAs in TCGA database. **J-Q** Differentially expressed 8 lncRNAs in TCGA database. **I-Y** Correlation between OS and 8 lncRNAs in TCGA database. “#” represents the lncRNAs whose correlation coefficient was less than − 0.15 in the miRNA-lncRNA Spearman test and was included in subsequent analysis

**Fig. 6:**
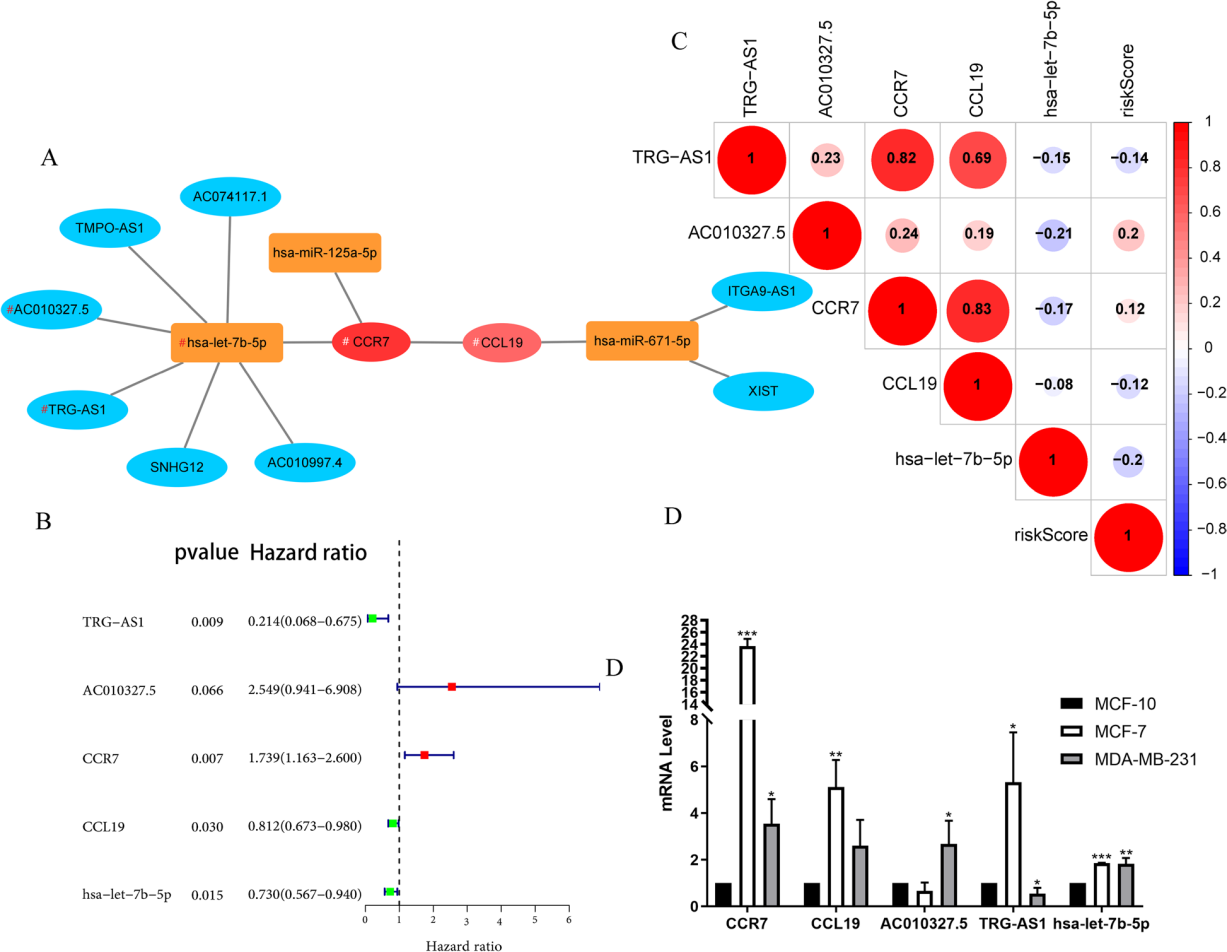
Construction of ceRNA network and Cox analysis. **A** The ceRNA network in BC visualized using Cytoscape 3.8.2 software. The red round nodes represent mRNAs, yellow rectangle nodes represent miRNAs, and the blue round nodes represent lncRNAs. **B** The forest map showed 5 RNAs identified by multivariate Cox analysis. **C** The relationship between 5 RNAs. **D** Detection of mRNA expression levels of 5 genes in MCF-10, MCF-7 and MDA-MB-231 cell lines using quantitative real-time PCR. (*P < 0.05, **P < 0.01, ***P < 0.001)

Secondly, we obtained 122 has-miR-125a-5p-lncRNA pairs, 145 has-let-7b-5p-lncRNA pairs and has-miR-671-5p-lncRNA pairs (Fig. 5A). 8 lncRNAs were obtained by the same co-expression analysis as above, including AC010327.5, AC010997.4, AC074117.1, ITGA9-AS1, SNHG12, TMPO-AS1, TRG-AS1 and XIST. Correlation analysis showed that these lncRNAs were inversely proportional to the three miRNAs (Fig. 5B-I). All of these 8 lncRNAs were highly expressed in tumor tissues (Fig. 5J-Q). In TCGA database, we found that OS differences between high and low expression groups of AC010997.4, TRG-AS1, SNHG12 and AC074117.1 by K-M test (Fig. 5I-Y). Finally, we merged the 3 mRNA-miRNA pairs and 8 miRNA-lncRNA pairs to construct a ceRNA network (Fig. 6A). We uploaded the data including id, survival time (futime), survival status (fustat) and expression of CCR7, CCL19, TRG-AS1, AC010327.5 and hsa-let-7b-5p as Supplement I.

### Establishment of Risk Model

We randomly divided the samples of BC patients into Train and Test groups, and analyzed the Train group, Test group and All group respectively. Firstly, multivariate Cox analysis was performed on 13 RNAs in Train group, and 5 RNAs were extracted by stepwise regression method and incorporated into Cox regression model. We found that `TRG-AS1`, CCL19 and `hsa-let-7b-5p` was a protective factor (HR < 1); AC010327.5 and CCR7 were risk factors (HR > 1) (Fig. 6B). Figure 6 C showed that the correlation between 5 RNAs and riskScore. TRG-AS1 and CCR7 (*r* = 0.82), TRG-AS1 and CCL19 (*r* = 0.63), CCR7 and CCL19 (*r* = 0.83) have high co-expression relationship. Figure 6D shows the RNA expression of these five genes in normal breast MCF-10 cell lines and breast cancer MCF-7 and MDA-MB-231 cell lines. The expression of CCR in MCF-7 was significantly higher than that in MCF-10 (*p* < 0.001).

### Validation of Risk model

**Fig. 7:**
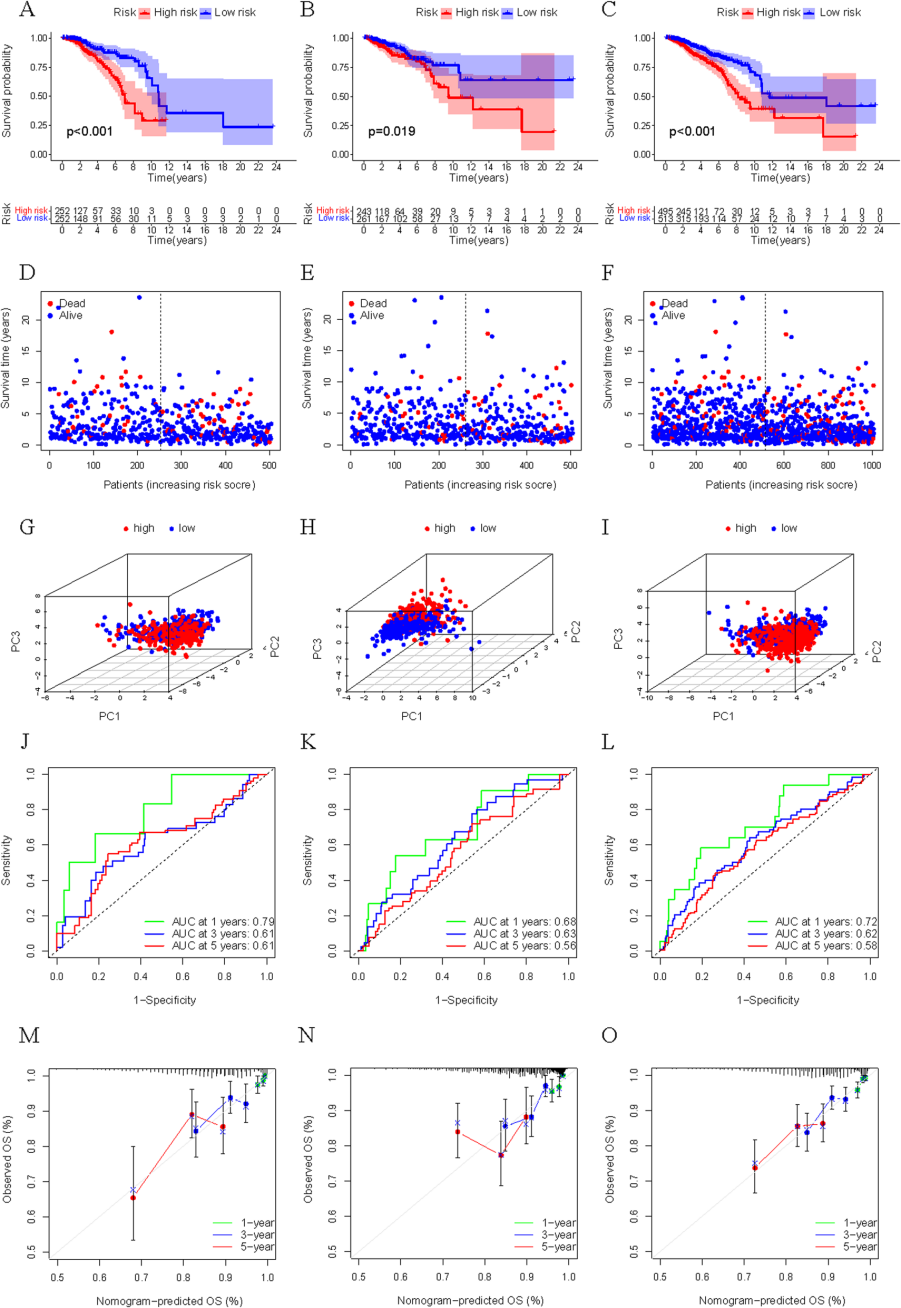
Validation of the risk model in Train, Test and All group. **A-C** Kaplan Meier curves of overall survival in the Train group (**A**), Test group (**B**) and All group (**C**). **D-F** OS status distribution of the high- or low-risk group in the Train group (**D**), Test group (**E**) and All group (**F**). **G-I** Principal components analysis between low- and high-risk groups in the Train group (**G**), Test group (H) and All group (**I**). **J**-**L** The correlation scatter plot between the riskScore and overall time in the Train group (**J**), Test group (K) and All group (**L**). **M-O** The 1-, 3- and 5-years receiver operating characteristic (ROC) curve analysis in the Train group (M), Test group (**N**) and All group (**O**). **P-I** The 1-, 3- and 5-years calibration curve in the Train group (**P**), Test group (**Q**) and All group (**I**)

Finally, the calculation formula of riskScore is as follows: riskScore= -1.544 *`TRG-AS1`+ 0.936 * AC010327.5 + 0.553 *CCR7 -0.208 *CCL19 -0.315 *`hsa-let-7b-5p. We then calculated the riskScore for each sample and divided patients into high-risk and low-risk groups based on the median riskScore for Train (Train group: 252 at high risk, 252 at low risk; Test group: 243 at high risk and 261 at low risk; All group: 495 at high risk, 513 at low risk). In the survival analysis, K-M curve showed that the survival rate of the low-risk group was significantly higher than that of the high-risk group (*p* < 0.05) (Fig. 7A-C). Figure 7D-F shows that the high-risk group had a higher rate of early death than the low-risk group. PCA was used to prove significant difference in distribution between low-risk and high-risk groups according to riskScore (Fig. 7G-I). To verify the accuracy of the model, ROC curves (Fig. 7J-L) and calibration curves (Fig. 7M-O) of 1-, 3- and 5-years were drawn, which showed that the model had good predictive power.

### Correlation with clinical trails

**Fig. 8:**
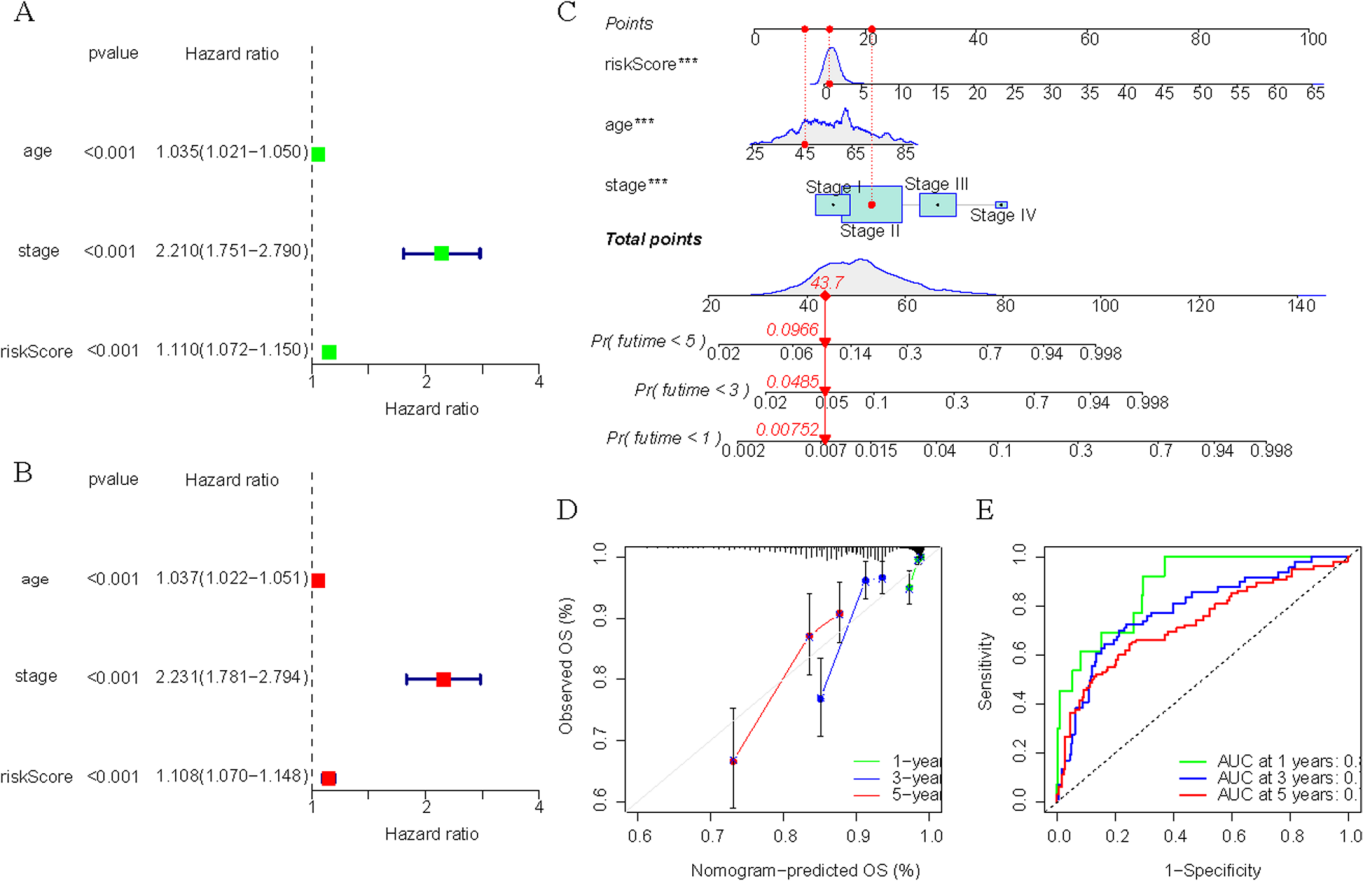
Correlation with clinicopathological features. **A**,** B** Univariate (**A**) and multivariate (**B**) Cox regression models indicated the riskScore was an independent prognostic factor. **C** The nomogram for predicting probabilities of BC patients’ overall survival. **D** ROC curve of 1-, 3-, and 5-year predictive power of clinical prognostic models. **E** calibration curve of 1-, 3-, and 5-year predictive power of clinical prognostic models

In order to explore the relationship between the riskScore and clinicopathological features of BC, we used riskScore as a variable in the All group and conducted univariate and multivariate Cox regression analyses in combination with other clinicopathological features (age and TNM stage). The results showed that age, stage and riskScore were independent risk factors for prognosis (HR > 1, *p* < 0.05) (Fig. 8A, B). In addition, these risk factors were used to construct a clinical prognosis model and plotted a Nomogram (Fig. 8C). In the nomogram, we randomly selected a patient, who was 45 years old, in stage II and whose total score was 43.7, thus estimating that the 5-year survival rate of the patient was 9.66%, 3-year survival rate was 4.85%, and 1-year survival rate was 0.752%. C-index was calculated as 0.7657. Meanwhile ROC curve and calibration curve illustrate the accuracy of the model (Fig. 8D, E).

### Enrichement analysis

**Fig. 9:**
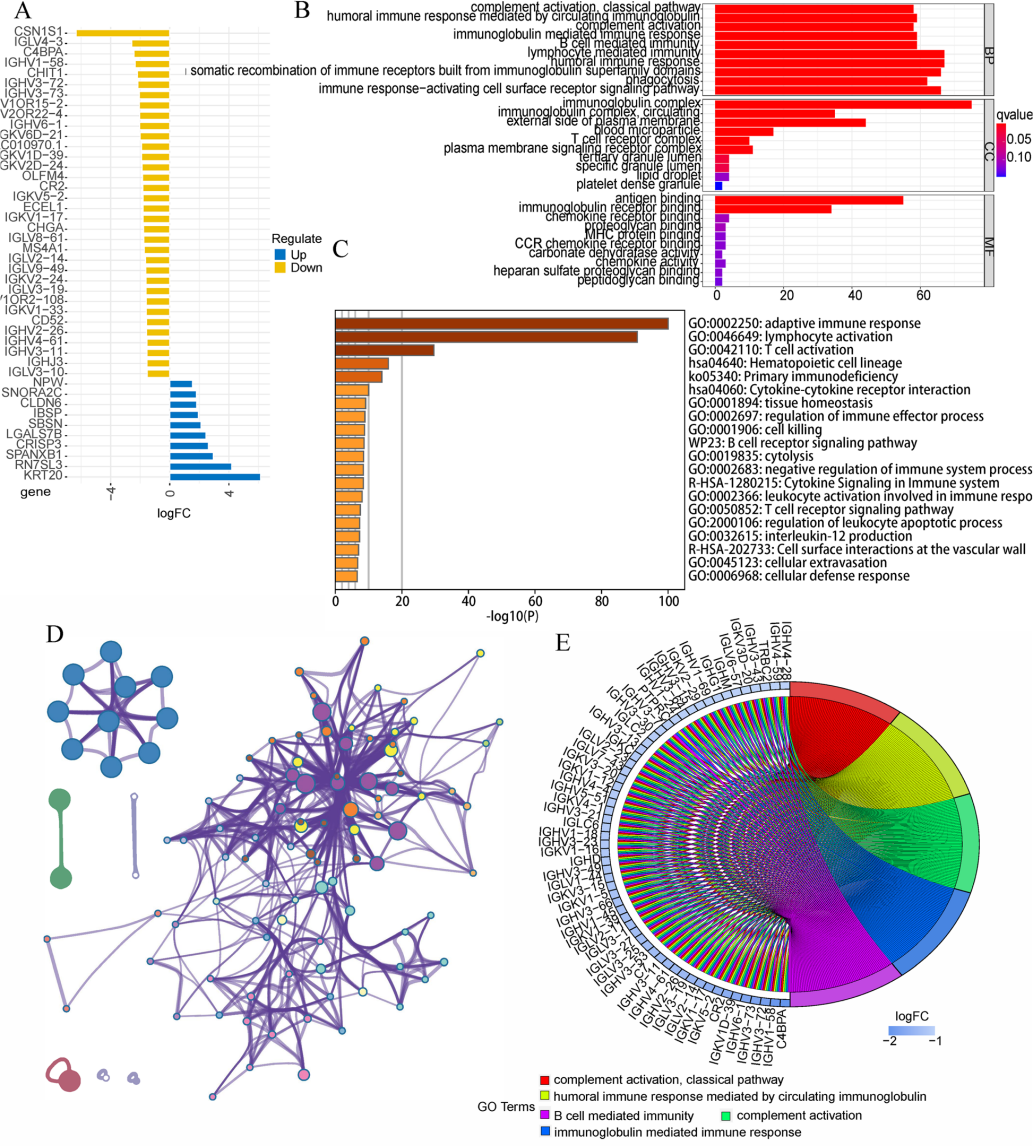
Functional enrichment analysis of risk difference genes. **A** Risk differential genes that meet the criteria of |logFC|>2. **B**, **E** GO analysis using R “org.Hs.eg.db” package [60–62]. **C**,** D** GO analysis in metascape online tool

**Fig. 10:**
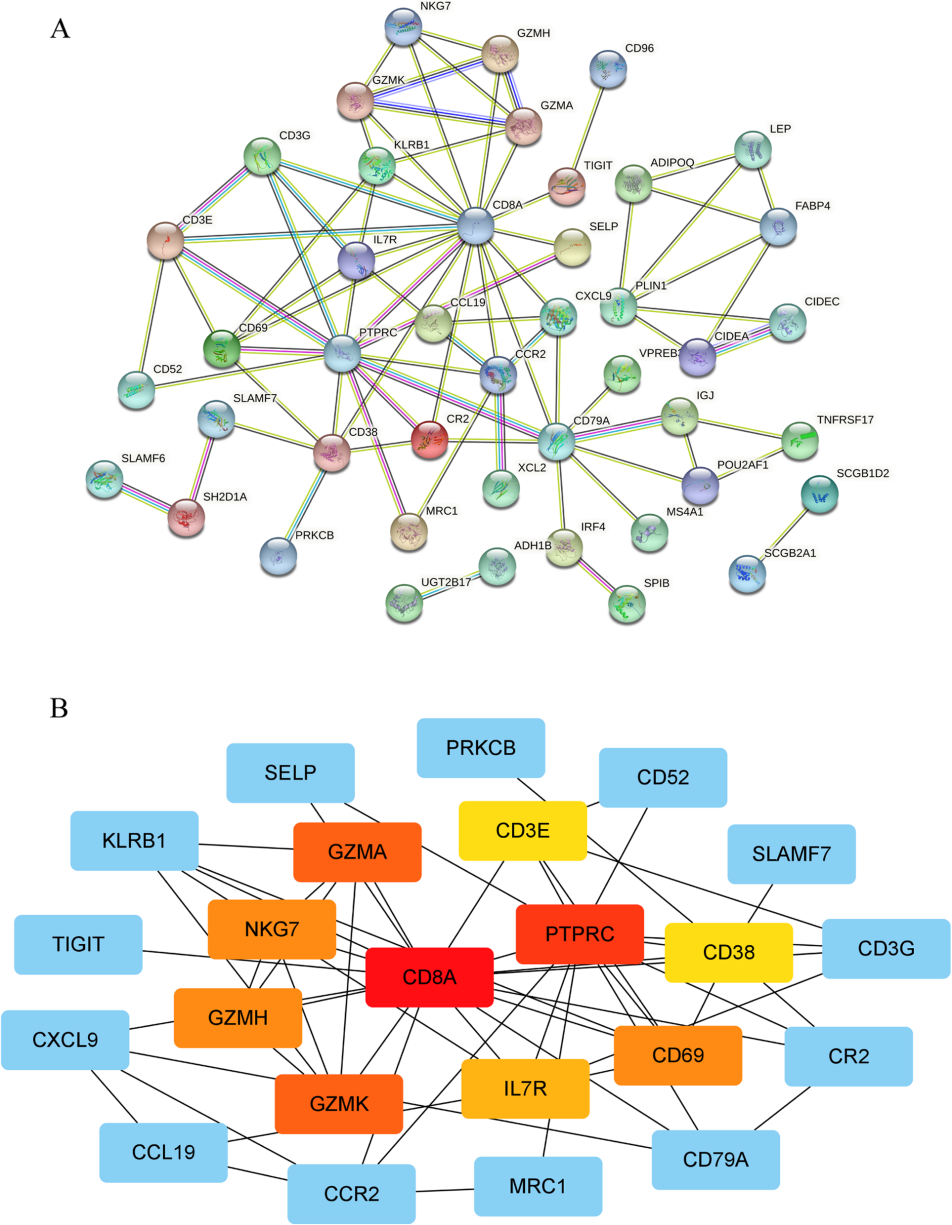
PPI networks of risk difference genes. **A** in STRING online tool. **B** in cytoscape software. The darker the color, the higher the degree score

Two hundred risk difference genes were found through analyzing the differences between high- or low-risk group of patients. Figure 9 A shows all the genes that meet the criteria of |logFC|>1.5. GO enrichment analysis showed that risk differential genes were mainly enriched in complement activation, classical pathway, humoral immune response mediated by circulating immunoglobulin, complement activation, immunoglobulin mediated immune response and B cell mediated immunity (Fig. 9B, E). Metascape online tool results showed that adaptive immune Response, B cell receptor signaling pathway and immunoglobulin production were closely related to risk differential genes (Fig. 9C, D). To investigate the interaction of risk differential genes, we mapped PPI networks using the STRING online tool and Cytoscape software (Fig. 10A). It can be seen from Fig. 10B that PTPRC and CD8A have the most related nodes.

### Important role in immune

Since enrichment analysis showed that the risk model was highly correlated with immunity, we further analyzed the correlation between the prognostic model and immune cell infiltration, tumor microenvironment, immune-related cells and pathways, and HLA genes in the Train group.

**Fig. 11:**
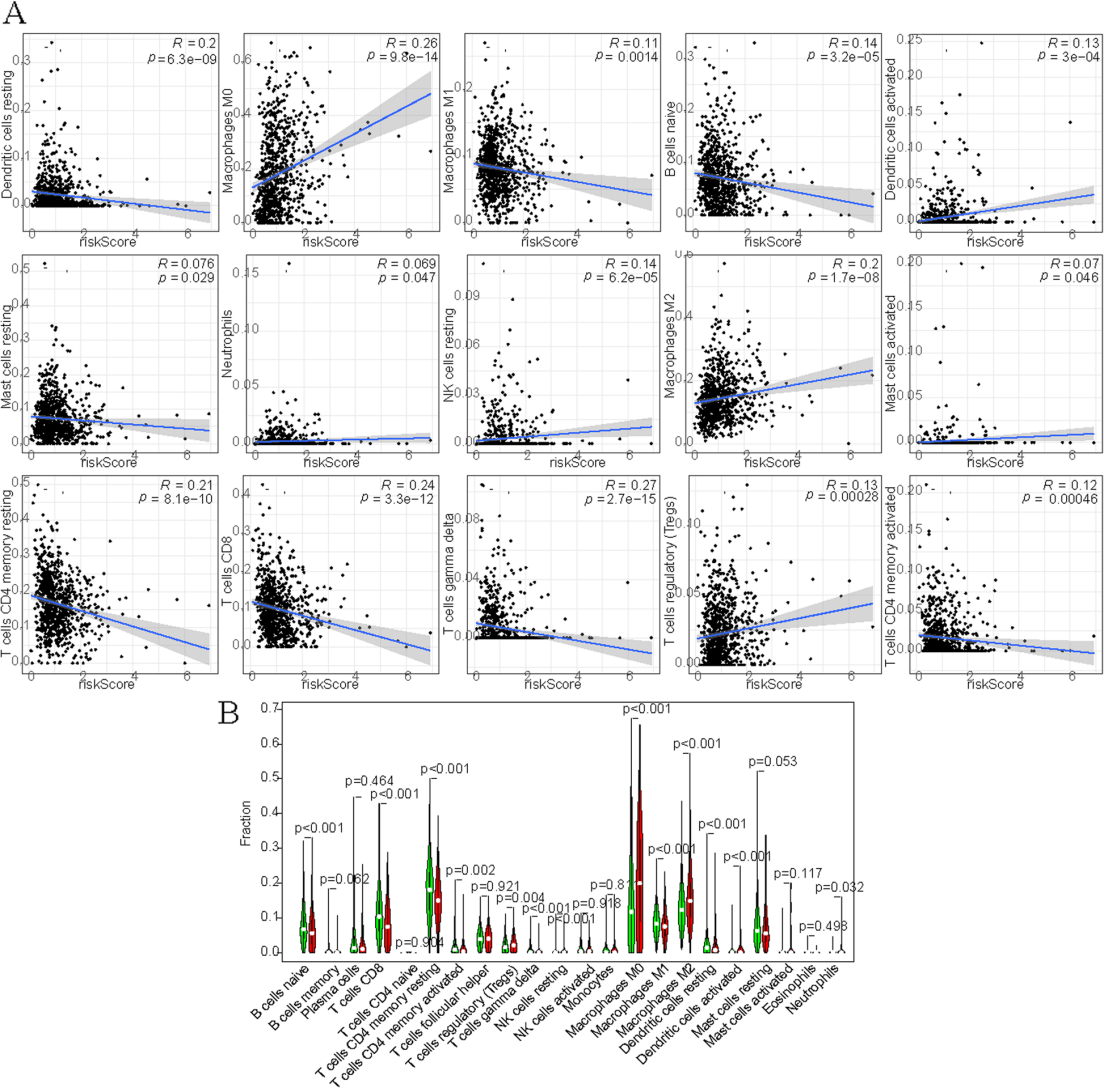
The correlation between immune infiltrating cells and risk model. **A** Correlation analysis between immune infiltrating cells and RiskScore. **B** Differences analysis in immune infiltrating cells between high and low-risk groups

First, CIBERSORT algorithm was used to calculate the score of immunoinfiltrating cells in each BC patient. In the differential analysis of immunoinfiltrating cells, we found significantly differences in B cells naïve, T cells CD8, T cells CD4 memory resting, Macrophages M0, etc. between the high and low-risk groups (*p* < 0.05) (Fig. 11B). Correlation analysis showed that T cells gamma delta, T cells CD8, etc. were negatively correlated with riskScore; Mast cells activated, Dendritic cells activated, etc. were positively correlated with riskScore (Fig. 11A).

**Fig. 12:**
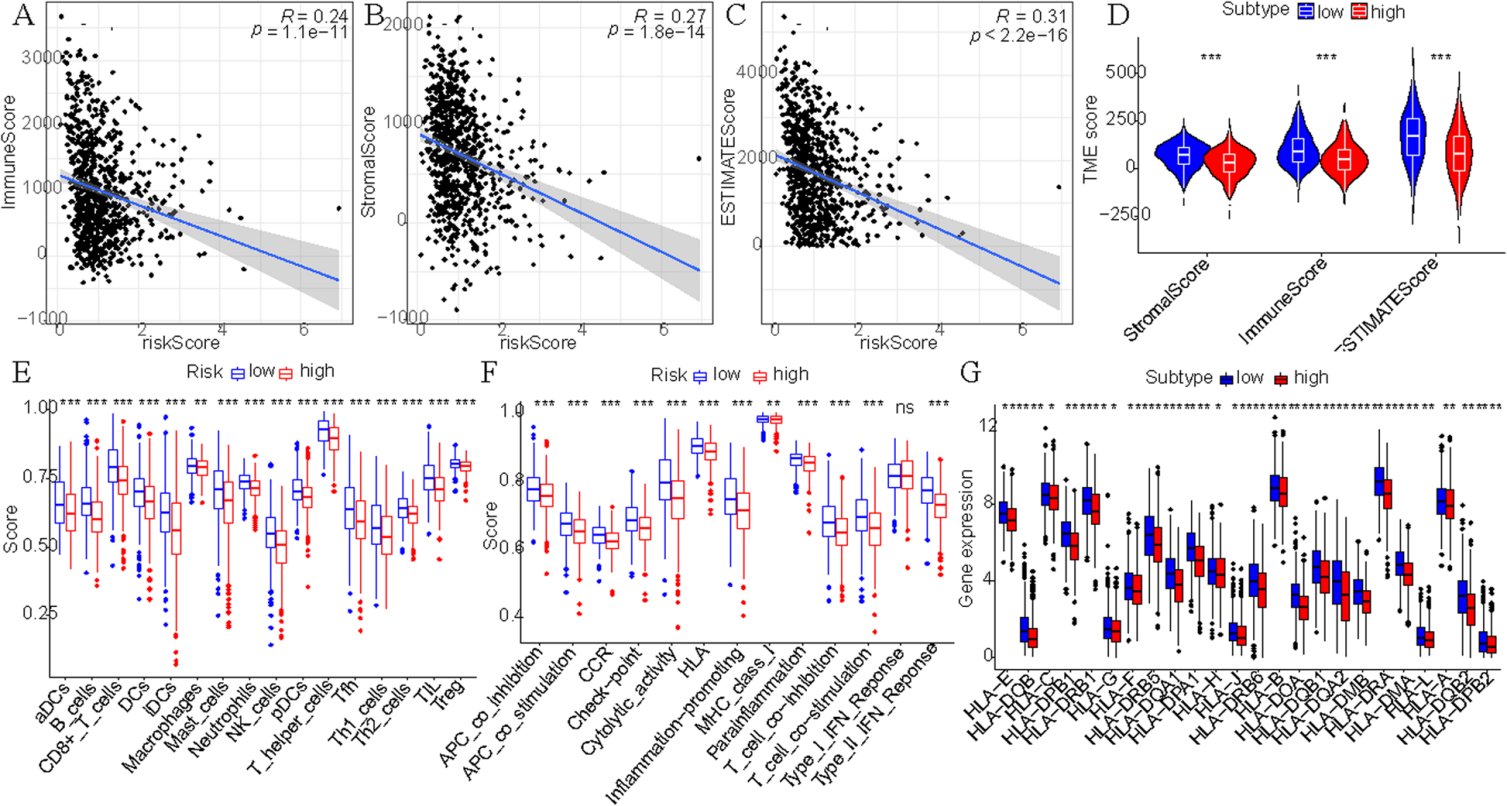
The important role in immunity. **A-C** Correlation analysis between tumor microenvironment score and riskScore. **D** Differences analysis in tumor microenvironment score between high and low-risk groups. **E** Differences analysis in immune-related cells between high and low-risk groups by ssGSEA. **F** Differences analysis in immune-related pathways between high and low-risk groups by ssGSEA. **G** Differences analysis in HLA genes between high and low-risk groups

Second, StromalScore, ImmuneScore, and ESTIMATEScore are calculated using the R “Estimate” package, which were respectively − 0.265278873, -0.235851355 and − 0.306087818 (Fig. 12A-C). Besides, all the scores were higher in the low-risk group than in the high-risk group (Fig. 12D).

Third, immune-related cells and pathway score were scored by ssGSEA analysis in each BC sample. In addition, there was no statistically significant difference in Type_I_IFN_Reponse between the high and low-risk groups, 12 of the immune pathways in the low-risk group had higher immune scores (Fig. 12E). As shown in Fig. 12F 16 immune cells were different between the high and low-risk groups, and the immune score of the low-risk group was higher than that of the high-risk group. The results were consistent with the correlation analysis and enrichment analysis of immune infiltrating cells.

Fourth, HLA plays a key role in the presentation of antigens to T cells and in the basic formation of host defense mechanisms against pathogens [13]. We found significant differences in HLA genes between high and low-risk groups (Fig. 12G).

Based on the above analysis, we conclude that this prognostic model plays an important role in immune-related functions and pathways.

### Drug sensitivity analysis by cMap

**Fig. 13:**
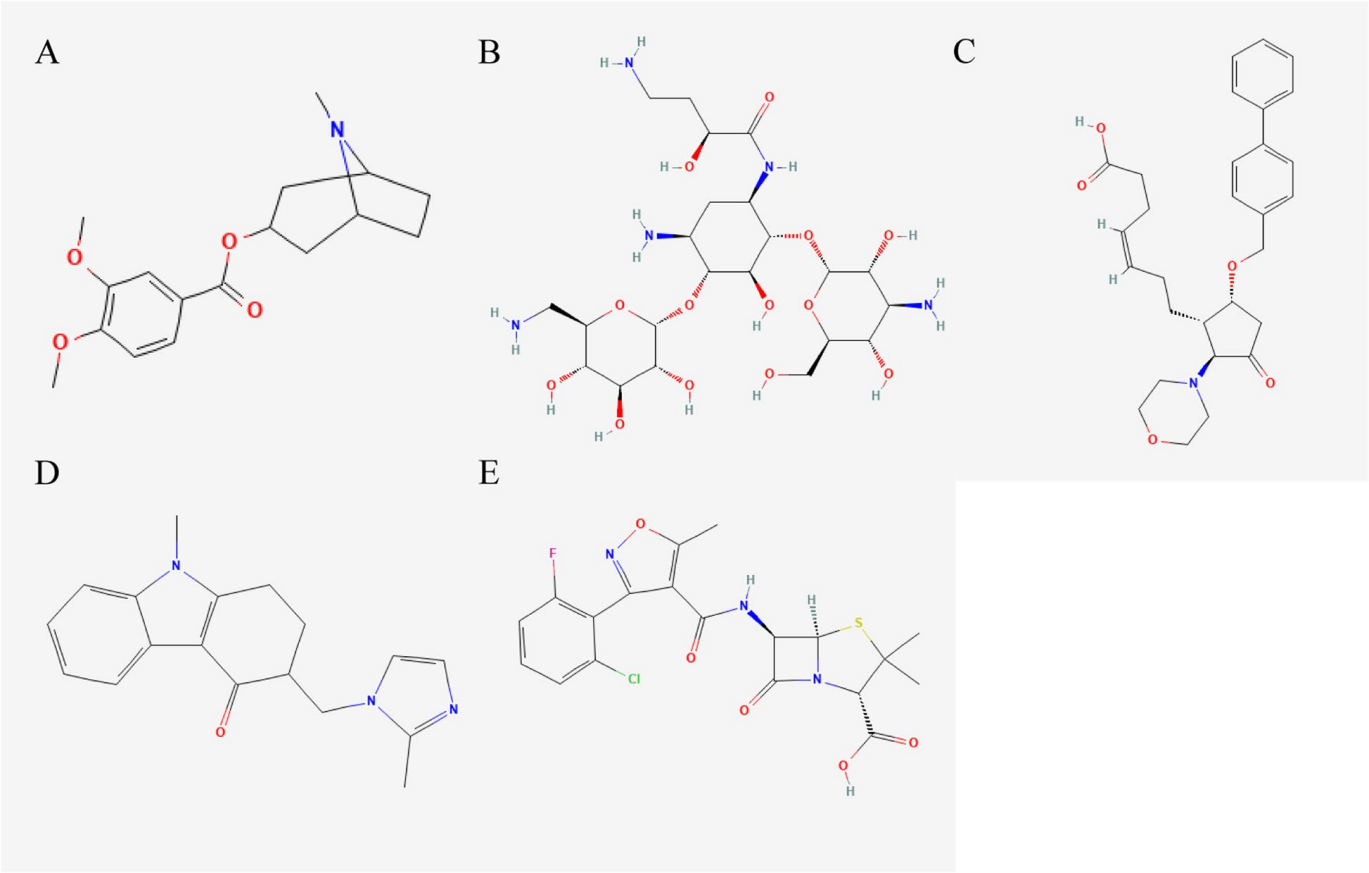
Molecular structures of 5 drugs found by cMap

Potential small molecule drugs were explored using cMap and totally 5 molecule drugs were screened out (Table 2). Megestrol (mean = -0.451, *n* = 4, *P* < 0.001) has a greater potential in the treatment of BC, whose molecular structure is shown in Fig. 13.

**Table 2:** Results of cMap analysis based on risk difference genes

cMap name	mean	n	enrichment	p	specificity	percent non-null
Convolamine	-0.573	4	-0.888	0.00036	0	75
Amikacin	0.465	4	0.864	0.00046	0.0089	75
AH-23,848	-0.76	3	-0.933	0.00048	0	100
Ondansetron	-0.442	4	-0.835	0.00133	0	50
Flucloxacillin	-0.412	4	-0.823	0.00189	0.0063	50

### Cellular activity and migration capacity

**Fig. 14:**
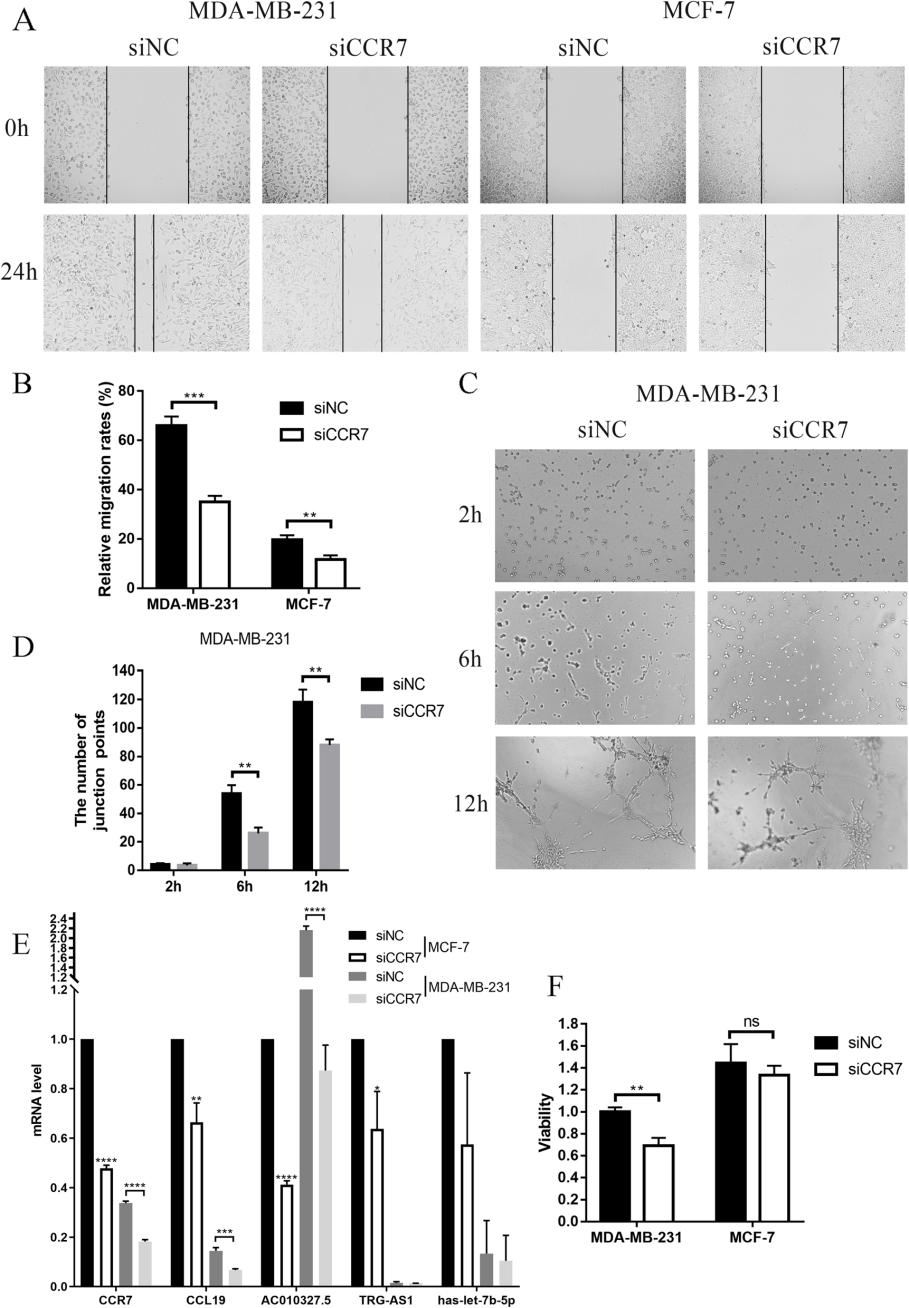
CCR7 regulates breast cancer cell migration and angiogenesis. **A**,** B** siCCR7 transfected MDA-MB-231 and MCF-7 cells, the effect of CCR7 on cell migration ability was assessed by scratch assay. **C**,** D** siCCR7 transfected MDA-MB-231 cells, the effect of CCR7 on tumour angiogenesis was assessed by angiogenesis assay. **E** siCCR7 transfected MDA-MB-231 and MCF-7 cells, the RNA expression levels of five prognostic genes were detected by PCR. **F** siCCR7 was transfected with MDA-MB-231 and MCF-7 cells, the effect of CCR7 on cellular activity was assessed by CCK8 assay. (*P < 0.05, **P < 0.01, ***P < 0.001, ****P < 0.0001)

To further explore the effect of CCR7 on breast cancer cells, we transfected MCF-7 and MDA-MB-231 cells using siCCR7. The results of the cell scratch assay showed that the migration ability of the cells was reduced after knockdown of CCR, and this phenomenon was more pronounced in MDA0MB0231 cells (Fig. 14A, B). CCR7 is a promoter of lymph angiogenesis and angiogenesis induction. Further angiogenesis assays showed a significant decrease in tubular tissue in MDA-MB-231 cells after knockdown of CCR7 at 12 h compared to control (Fig. 14C, D). CCK-8 results showed that the cellular activity of siCCR7 group was significantly lower than that of siNC group in MDA-MB-231 cells (Fig. 14F). PCR results showed that CCL19 and AC010327.5 RNA expression was reduced along with knockdown of CCR7 (Fig. 14E).

## Discussion

Breast cancer is the most common malignant tumor in women, which is a serious threat to women’s health and life. According to the latest global cancer burden data for 2020 released by the International Agency for Research on Cancer (IARC), the number of new cases of BC is growing rapidly worldwide and has replaced lung cancer as the number one cancer worldwide [14]. The development and progression of BC is due to a combination of factors, and its high degree of heterogeneity depends largely on the activation and loss of its proto-oncogenes, oncogenes and signaling pathways [15]. The expression of the relevant genes can indirectly reflect the proliferation ability of cancer cells and assess the malignancy of the tumor. Therefore, it is urgent to find sensitive and specific indicators at the molecular level to assess the progression and prognosis of BC survival. An increasing number of studies have found that some chemokines and their receptors are highly expressed on the surface of tumor cells, which are involved in the metastatic process of tumors [16]. And the migration and metastasis of BC tumors depend largely on the interaction between chemokine receptors and chemokines expressed in cancer cells and metastatic disease sites.

CCR7 is important for tissue homeostasis, immune surveillance, and tumorigenesis through binding to chemokines CCL19 and CCL21. CCR7/CCL19 promotes the development of systemic tumors such as BC [17], pancreatic cancer [18], melanoma [19], non-small cell lung cancer [20] and gastric cancer [21], and mediates the movement of dendritic cells to lymphoid organs playing an important role in the immune response and the transport and homing of lymphocytes to lymph nodes [22–24]. Bing Xu et al. found that CCL19 activates AKT signaling pathway to mediate tumor cell invasion and migration and regulates epithelial-mesenchymal transition (EMT) process of BC cells through CCR7/CCL19 axis [25, 26]. The binding of CCR7 and its ligand CCL19 induces chemotactic migration and cytoskeletal rearrangement of target cells, which are involved in a variety of physiological and pathological processes that regulate tumor survival and proliferation, invasion, and metastasis, including leukemia [27] and head and neck tumors [26]. CXCR4 has been shown to be an important prognostic marker in BC [28]. In cancer CCR7 is upregulated together with CXCR4, and the dimer formed by both can activate signaling pathways and promote tumor metastasis [28]. Hayasaka et al. examined the effect of CXCL12 on the CCR7-dependent signaling in MDA-MB-231 human BC cells and found that CXCL12 promotes homodimer formation, ligand binding, and cellular responses at lower concentrations of CCL19 [29]. *It has also been found that CCL19 can increase the heterogeneity of breast cancer cell motility in the 3D microenvironment of breast cancer. Cell motility was more asymmetric at CCL19 concentrations close to the CCR7 dynamic kinetic binding constant, suggesting that CCL19 is involved in regulating tumor cell heterogeneity and invasive ability in the breast cancer microenvironment* [30]. *CCL19 in breast cancer is considered an important biological marker for tumor diagnosis and prognosis* [31].

*In previous reports in the literature, tumor-associated mRNAs were always screened by differential analysis, which lacked biological plausibility. Potential mechanisms underlying breast cancer prognosis from an immunochemokine perspective are unknown. Based on the fact that CCR7/CCL19 plays a crucial role in breast cancer development and metastasis, we further explored the IncRNAs and micRNAs associated with it and constructed a ceRNA network. And the risk prognosis model was constructed by multifactor Cox analysis. Models included CCR7, CCL19, TRG-AS1, AC010327.5 and hsa-let-7b-5p. LncRNA TRG-AS1 promotes the ability of some tumor cells to proliferate, differentiate, epithelial mesenchymal transition, migration, invasion and signaling. As a highly conserved regulatory factor, LncRNA TRG-AS1 does not have the function of encoding proteins per se, but it can directly interact with a variety of transcription factors in the form of RNA to precisely regulate the expression of target genes and influence tumor progression* [32, 33]. *Let-7, the largest known mi RNA family, is conserved across multiple species and plays a role in the development of a variety of epithelial tumors. most of the miRNAs in the Let-7 family, except let-7a-3, are oncogenes, which are involved in oncocyte cycle regulation and affect cancer cell migration and invasion. Numerous studies have shown that hsa-let-7b-5p functions as a tumor suppressor in cervical cancer, breast cancer and glioma* [34, 35].RiskScore combines clinical factors (age and stage) to further diagnose the survival risk of BC patients for clinicians. Some studies reported [36] that TNM stage and tumor size differed in metastatic and non-metastatic BC (*P* < 0.001), suggesting that our model may be a good predictor of tumor metastasis.

We genetically enriched 200 risk differential genes and found that it was mainly enriched in complement activation, immune response mediated by immunoglobulin, B cell and lymphocyte. Cuesta-Mateos et al. [37] demonstrated that anti-CCR7 monoclonal antibodies can preserve T cell subsets while effectively removing tumor cells through a complement-mediated mechanism of action in chronic lymphocytic leukemia (CLL). CD4 + CXCR5 + TH cells can migrate to B lymphocytes and contribute to immunoglobulin type switching by downregulating CCR7 expression [38]. Qin et al. [39] found that in bone marrow Mesenchymal Stem Cells (MSCs) the proliferation of CD19 + IL-10 + Breg cells could be directly promoted through the SDF-1α/CXC4R and CCL19/21-CCR7 axes thereby suppressing the immune microenvironment. CCR7/CCL21/CCL19 are key molecules that regulate lymphocyte homing and promote tumorigenic development and pleiotropic effects [40]. Mature DCs and most naive T lymphocytes express CCR7 [41], which can complete homing in response to the concentration gradient of cytokines CCL19 and CCL21 secreted by secondary lymphoid organs [42].

We found 2 Hub genes, CD8A and PTPRC, in the PPI network diagram. CD8A is a marker of CD8 + T cells. Zhang et al. also identified CD8A as one of the top 10 hub genes by bioinformatics analysis [43]. CCR7 with its ligands CCL21 and CCL19 regulates the apoptosis of CD8 + T cells and participates in the process of tumor microenvironment remodeling [44]. Receptor-type tyrosine-protein phospha-tase C (PTPRC) is involved in natural killer cell-mediated cytotoxic responses, chemokine signaling pathways, T-cell receptor signaling and cytokine-receptor interactions in the inflammatory response [45], and is closely related to the CCR7 chemokine axis.

To further explore the relationship between the CCR7/CCL19 chemokine axis prognostic risk model and immunity, we explored the relationship between riskScores and immune infiltrating cells, tumor microenvironment, ssGSEA scores and HLA genes. Cellular chemokines are important modulators of immune cell infiltration and inflammatory responses [46], which can undergo recruitment to infiltrate immune cells into the tumor. The CCR7/CCL19 chemokine axis plays an important role in mediating immune cells against tumors [47]. Meanwhile, CCL21/CCL19 attract CCR7 + T cells as well as other immune cells and alter the ectopic lymph node structure associated with cancer prognosis by co-localizing synaptic cells and T cells, thereby promoting immune activity in the tumor microenvironment (TME) leading to immune infiltration [48]. Iida et al. [49] enhanced the therapeutic effect of anti-PD-L1 antibodies by promoting immune cell infiltration through local injection of CCL19 mesenchymal stem cells (iMSC/CCL19) into mice. HLA-F expression is associated with the ability of tumor cells to escape the body’s immune killing capacity, and its reduced expression suggests metastasis of tumor cells [50].

In the drug sensitivity analysis, we found five drugs (convolamine, amikacin, AH-23,848, ondansetron, and flucloxacillin). Convolamine contains many bioactive phytoconstituents, such as alkaloids, flavonoids and phenolics, which can modulate immunotherapy [51]. Amikacin is an aminoglycoside antibiotic. Yun-Hsin et al. [52] found that amikacin inhibited the migration of human BC MDA-MB-231 cells [52]. AH-23,848 is an EP4 antagonist and cyclooxygenase 2 (COX-2) can stimulate CCR7 expression through EP2/EP4 receptors, thereby promoting lymphatic invasion of BC cells [53, 54]. In a randomized double-blind controlled trial, Olanzapine combined with ondansetron and dexamethasone was more effective than placebo in preventing chemotherapy-induced nausea and vomiting (CINV) caused by doxorubicin plus cyclophosphamide in patients with early-stage BC, especially in the first 24 h after chemotherapy [55]. Flucloxacillin is an antimicrobial resistance to penicillinase and is designed for oral and injectable administration with bactericidal activity [56]. These five drugs have been relatively little studied in BC and need further research.

 Finally, we found that CCR7 was associated with cell migration and angiogenesis by knocking down CCR7 expression in breast cancer cells. Studies have reported that CCR7 can be involved in various biological behaviors of tumor cells, such as proliferation, migration, and angiogenesis, and plays an important role in them [57]. It has been shown that CCR7 is expressed in the vascular endothelium surrounding tumors and plays an important role in angiogenesis [58]. CCR7 can regulate the migration of T cells and dendritic cells, chemotaxis of immune cells to target organs, and also regulate their immune function, causing inflammatory responses [59].

In conclusion, we constructed the first prognostic model based on the CCR7/CCL19 chemokine axis in BC and explored its role in immune infiltration, tumor microenvironment, and HLA genes. This study provides the biological basis for an in-depth study of the role of CCR7 chemokine week in BC and identifies new drugs for the possible treatment of BC through drug sensitivity analysis. We also knocked down CCR7 in MDA-MB-231 and MCF-7 cells and observed changes in cell activity and migratory capacity.

### Supplementary Information

**Additional file  1: Supplement 1. **Survival data and RNA expression in the TCGA-BRCA database.

## Data Availability

The original contributions presented in the study are included in the article/Additional files, further inquiries can be directed to the corresponding authors.

## References

[1] Ginsburg O, Bray F, Coleman MP, Vanderpuye V, Eniu A, Kotha SR (2017). The global burden of women’s cancers: a grand challenge in global health. Lancet (London England).

[2] Hughes CE, Nibbs RJB (2018). A guide to chemokines and their receptors. FEBS J.

[3] Xia X, Liu K, Zhang H, Shang Z (2015). Correlation between CCR7 expression and lymph node metastatic potential of human tongue carcinoma. Oral Dis.

[4] Schweickart VL, Raport CJ, Godiska R, Byers MG, Eddy RL, Shows TB (1994). Cloning of human and mouse EBI1, a lymphoid-specific G-protein-coupled receptor encoded on human chromosome 17q12-q21.2. Genomics.

[5] Liu Y, Ji R, Li J, Gu Q, Zhao X, Sun T (2010). Correlation effect of EGFR and CXCR4 and CCR7 chemokine receptors in predicting breast cancer metastasis and prognosis. J Experimental Clin cancer Research: CR.

[6] Wick N, Saharinen P, Saharinen J, Gurnhofer E, Steiner CW, Raab I (2007). Transcriptomal comparison of human dermal lymphatic endothelial cells ex vivo and in vitro. Physiol Genom.

[7] Yoshida R, Imai T, Hieshima K, Kusuda J, Baba M, Kitaura M (1997). Molecular cloning of a novel human CC chemokine EBI1-ligand chemokine that is a specific functional ligand for EBI1, CCR7. J Biol Chem.

[8] Salmena L, Poliseno L, Tay Y, Kats L, Pandolfi PP (2011). A ceRNA hypothesis: the Rosetta Stone of a hidden RNA. Language? Cell.

[9] Jiang D, He Y, Mo Q, Liu E, Li X, Huang L (2021). PRICKLE1, a Wnt/PCP signaling component, is overexpressed and associated with inferior prognosis in acute myeloid leukemia. J Translational Med.

[10] Li JH, Liu S, Zhou H, Qu LH, Yang JH (2014). starBase v2.0: decoding miRNA-ceRNA, miRNA-ncRNA and protein-RNA interaction networks from large-scale CLIP-Seq data. Nucleic Acids Res.

[11] Yoshihara K, Shahmoradgoli M, Martínez E, Vegesna R, Kim H, Torres-Garcia W (2013). Inferring tumour purity and stromal and immune cell admixture from expression data. Nat Commun.

[12] Jia Q, Wu W, Wang Y, Alexander PB, Sun C, Gong Z (2018). Local mutational diversity drives intratumoral immune heterogeneity in non-small cell lung cancer. Nat Commun.

[13] Wong LP, Ong RT, Poh WT, Liu X, Chen P, Li R (2013). Deep whole-genome sequencing of 100 southeast asian Malays. Am J Hum Genet.

[14] Siegel RL, Miller KD, Jemal A (2020). Cancer statistics, 2020. Cancer J Clin.

[15] Polyak K (2007). Breast cancer: origins and evolution. J Clin Investig.

[16] Balkwill F (2003). Chemokine biology in cancer. Semin Immunol.

[17] Szekely B, Bossuyt V, Li X, Wali VB, Patwardhan GA, Frederick C (2018). Immunological differences between primary and metastatic breast cancer. Annals of Oncology: Official Journal of the European Society for Medical Oncology.

[18] Nakata B, Fukunaga S, Noda E, Amano R, Yamada N, Hirakawa K (2008). Chemokine receptor CCR7 expression correlates with lymph node metastasis in pancreatic cancer. Oncology.

[19] Cristiani CM, Turdo A, Ventura V, Apuzzo T, Capone M, Madonna G (2019). Accumulation of circulating CCR7 natural killer cells Marks Melanoma Evolution and reveals a CCL19-Dependent metastatic pathway. Cancer Immunol Res.

[20] Baran K, Kiszałkiewicz J, Migdalska-Sęk M, Jabłoński S, Kordiak J, Antczak A (2019). An assessment of the relationship between the expression of CCR7/CCL19 axis and selected regulatory miRNAs in non-small cell lung cancer. Mol Biol Rep.

[21] Zhou R, Sun J, He C, Huang C, Yu H (2020). CCL19 suppresses gastric cancer cell proliferation, migration, and invasion through the CCL19/CCR7/AIM2 pathway. Hum Cell.

[22] Riol-Blanco L, Sánchez-Sánchez N, Torres A, Tejedor A, Narumiya S, Corbí AL (2005). The chemokine receptor CCR7 activates in dendritic cells two signaling modules that independently regulate chemotaxis and migratory speed. J Immunol (Baltimore, Md: 1950).

[23] Cheng KW, Lahad JP, Kuo WL, Lapuk A, Yamada K, Auersperg N (2004). The RAB25 small GTPase determines aggressiveness of ovarian and breast cancers. Nat Med.

[24] Le Y, Zhou Y, Iribarren P, Wang J (2004). Chemokines and chemokine receptors: their manifold roles in homeostasis and disease. Cell Mol Immunol.

[25] Xu B, Zhou M, Qiu W, Ye J, Feng Q (2017). CCR7 mediates human breast cancer cell invasion, migration by inducing epithelial-mesenchymal transition and suppressing apoptosis through AKT pathway. Cancer Med.

[26] Wang J, Seethala RR, Zhang Q, Gooding W, van Waes C, Hasegawa H (2008). Autocrine and paracrine chemokine receptor 7 activation in head and neck cancer: implications for therapy. J Natl Cancer Inst.

[27] Hoellenriegel J, Meadows SA, Sivina M, Wierda WG, Kantarjian H, Keating MJ (2011). The phosphoinositide 3’-kinase delta inhibitor, CAL-101, inhibits B-cell receptor signaling and chemokine networks in chronic lymphocytic leukemia. Blood.

[28] Molnár IA, Molnár BÁ, Vízkeleti L, Fekete K, Tamás J, Deák P (2017). Breast carcinoma subtypes show different patterns of metastatic behavior. Virchows Arch.

[29] Hayasaka H, Yoshida J, Kuroda Y, Nishiguchi A, Matsusaki M, Kishimoto K (2022). CXCL12 promotes CCR7 ligand-mediated breast cancer cell invasion and migration toward lymphatic vessels. Cancer Sci.

[30] Kim BJ, Hannanta-Anan P, Ryd A, Swartz MA, Wu M (2020). Lymphoidal chemokine CCL19 promoted the heterogeneity of the breast tumor cell motility within a 3D microenvironment revealed by a Lévy distribution analysis. Integr Biology: Quant Biosci nano Macro.

[31] Gowhari Shabgah A, Al-Obaidi ZMJ, Sulaiman Rahman H, Kamal Abdelbasset W, Suksatan W, Bokov DO (2022). Does CCL19 act as a double-edged sword in cancer development?. Clin Exp Immunol.

[32] Sun X, Qian Y, Wang X, Cao R, Zhang J, Chen W (2020). LncRNA TRG-AS1 stimulates hepatocellular carcinoma progression by sponging miR-4500 to modulate BACH1. Cancer Cell Int.

[33] Xu H, Zhou M, Cao Y, Zhang D, Han M, Gao X (2019). Genome-wide analysis of long noncoding RNAs, microRNAs, and mRNAs forming a competing endogenous RNA network in repeated implantation failure. Gene.

[34] Xi X, Chu Y, Liu N, Wang Q, Yin Z, Lu Y (2019). Joint bioinformatics analysis of underlying potential functions of hsa-let-7b-5p and core genes in human glioma. J Translational Med.

[35] Lirussi L, Ayyildiz D, Liu Y, Montaldo NP, Carracedo S, Aure MR (2022). A regulatory network comprising let-7 miRNA and SMUG1 is associated with good prognosis in ER + breast tumours. Nucleic Acids Res.

[36] Mohammed MM, Shaker O, Ramzy MM, Gaber SS, Kamel HS (2021). Abed El Baky MF. The relation between ACKR4 and CCR7 genes expression and breast cancer metastasis. Life Sci.

[37] Cuesta-Mateos C, Brown JR, Terrón F, Muñoz-Calleja C (2021). Of Lymph Nodes and CLL cells: deciphering the role of CCR7 in the pathogenesis of CLL and understanding its potential as therapeutic target. Front Immunol.

[38] Arnold CN, Campbell DJ, Lipp M, Butcher EC (2007). The germinal center response is impaired in the absence of T cell-expressed CXCR5. Eur J Immunol.

[39] Qin Y, Zhou Z, Zhang F, Wang Y, Shen B, Liu Y (2015). Induction of Regulatory B-Cells by mesenchymal stem cells is affected by SDF-1α-CXCR7. Cell Physiol Biochem.

[40] Pahne-Zeppenfeld J, Schröer N, Walch-Rückheim B, Oldak M, Gorter A, Hegde S (2014). Cervical cancer cell-derived interleukin-6 impairs CCR7-dependent migration of MMP-9-expressing dendritic cells. Int J Cancer.

[41] Sallusto F, Mackay CR, Lanzavecchia A (2000). The role of chemokine receptors in primary, effector, and memory immune responses. Annu Rev Immunol.

[42] Luther SA, Tang HL, Hyman PL, Farr AG, Cyster JG (2000). Coexpression of the chemokines ELC and SLC by T zone stromal cells and deletion of the ELC gene in the plt/plt mouse. Proc Natl Acad Sci USA.

[43] Zhang C, Peng L, Zhang Y, Liu Z, Li W, Chen S (2017). The identification of key genes and pathways in hepatocellular carcinoma by bioinformatics analysis of high-throughput data. Med Oncol (Northwood Lond Engl).

[44] Sánchez-Sánchez N, Riol-Blanco L, de la Rosa G, Puig-Kröger A, García-Bordas J, Martín D (2004). Chemokine receptor CCR7 induces intracellular signaling that inhibits apoptosis of mature dendritic cells. Blood.

[45] Rudnicki M, Perco P, Leierer BDH, Heinzel J, Mühlberger A (2016). Renal microRNA- and RNA-profiles in progressive chronic kidney disease. Eur J Clin Invest.

[46] Darling NJ, Arthur JSC, Cohen P (2021). Salt-inducible kinases are required for the IL-33-dependent secretion of cytokines and chemokines in mast cells. J Biol Chem.

[47] Takanami I (2003). Overexpression of CCR7 mRNA in nonsmall cell lung cancer: correlation with lymph node metastasis. Int J Cancer.

[48] Sharma S, Kadam P, Dubinett S (2020). CCL21 Programs Immune Activity in Tumor Microenvironment. Adv Exp Med Biol.

[49] Iida Y, Yoshikawa R, Murata A, Kotani H, Kazuki Y, Oshimura M (2020). Local injection of CCL19-expressing mesenchymal stem cells augments the therapeutic efficacy of anti-PD-L1 antibody by promoting infiltration of immune cells. J Immunother Cancer..

[50] Koyro TF, Kraus E, Lunemann S, Hölzemer A, Wulf S, Jung J (2021). Upregulation of HLA-F expression by BK polyomavirus infection induces immune recognition by KIR3DS1-positive natural killer cells. Kidney Int.

[51] Balkrishna A, Thakur P, Varshney A (2020). Phytochemical Profile, pharmacological attributes and Medicinal Properties of - A cognitive enhancer Herb for the management of neurodegenerative etiologies. Front Pharmacol.

[52] Wang YH, Chen YH, Shen WH (2020). Amikacin suppresses human breast Cancer Cell MDA-MB-231 Migration and Invasion. Toxics..

[53] Chuang C-W, Pan M-R, Hou M-F, Hung W-C (2013). Cyclooxygenase-2 up-regulates CCR7 expression via AKT-mediated phosphorylation and activation of Sp1 in breast cancer cells. J Cell Physiol.

[54] Pan M-R, Hou M-F, Chang H-C, Hung W-C (2008). Cyclooxygenase-2 up-regulates CCR7 via EP2/EP4 receptor signaling pathways to enhance lymphatic invasion of breast cancer cells. J Biol Chem.

[55] Tienchaiananda P, Nipondhkit W, Maneenil K, Sa-Nguansai S, Payapwattanawong S, Laohavinij S (2019). A randomized, double-blind, placebo-controlled study evaluating the efficacy of combination olanzapine, ondansetron and dexamethasone for prevention of chemotherapy-induced nausea and vomiting in patients receiving doxorubicin plus cyclophosphamide. Ann Palliat Med.

[56] de Menezes MNd BA, Fiorentino FAM, Zimmermann A, Kogawa AC, Salgado HRN, Flucloxacillin (2019). A review of characteristics, Properties and Analytical Methods. Crit Rev Anal Chem.

[57] Ağın A, Kiratli H, Guresci S, Babaoglu B, Karakaya J, Soylemezoglu F (2022). Evaluation of HSP-27, BAP1, BRAF V600E, CCR7, and PD-L1 expression in uveal melanoma on enucleated eyes and metastatic liver tumors. Int J Biol Mark.

[58] Zhang Q, Sun L, Yin L, Ming J, Zhang S, Luo W (2013). CCL19/CCR7 upregulates heparanase via specificity protein-1 (Sp1) to promote invasion of cell in lung cancer. Tumour Biology: The Journal of the International Society for Oncodevelopmental Biology and Medicine.

[59] Kobayashi D, Endo M, Ochi H, Hojo H, Miyasaka M, Hayasaka H (2017). Regulation of CCR7-dependent cell migration through CCR7 homodimer formation. Sci Rep.

[60] Kanehisa M, Goto S (2000). KEGG: kyoto encyclopedia of genes and genomes. Nucleic Acids Res.

[61] Kanehisa M (2019). Toward understanding the origin and evolution of cellular organisms. Protein Science: A Publication of the Protein Society.

[62] Kanehisa M, Furumichi M, Sato Y, Kawashima M, Ishiguro-Watanabe M (2023). KEGG for taxonomy-based analysis of pathways and genomes. Nucleic Acids Res.

